# Reducing Long-Term Clozapine Blood Monitoring Frequency: A Service Evaluation of Safety, Acceptability and Patient Experience

**DOI:** 10.1192/bjo.2026.11604

**Published:** 2026-06-30

**Authors:** Mihai Pop, Haitham Ismail, Susham Gupta, Andrea Okoloekwe

**Affiliations:** East London NHS Foundation Trust, London, United Kingdom

## Abstract

**Aims:**

To evaluate the safety, feasibility and acceptability of reducing clozapine blood monitoring frequency from monthly to three-monthly in patients established on clozapine for at least one year, while maintaining monthly physical health reviews.

**Methods:**

Clozapine is the most effective treatment for treatment-resistant schizophrenia but remains underutilised in the United Kingdom (UK). One of the key barriers to clozapine continuation is the stringent long term haematological monitoring. A review of the literature examined the incidence and timing of clozapine-associated neutropenia and agranulocytosis, international monitoring practices and findings from services using reduced monitoring schedules. The literature suggests that the risk of clozapine-induced agranulocytosis is highest within the first year of treatment, raising concerns that current UK guidelines may be overly restrictive beyond this point. The review findings guided the implementation of a pilot service evaluation at East London NHS Foundation Trust. Between May and September 2025, 32 patients meeting eligibility criteria were enrolled. Eligibility criteria were adults aged 18 to 65 years, stable on clozapine for more than one year, with capacity to consent to less frequent monitoring and awareness of infection symptoms. Participants received full blood count monitoring every three months alongside ongoing monthly physical health reviews and medication supply. Safety outcomes included haematological results and clinic attendance. Patient and staff experience were assessed monthly using a five-point satisfaction scale (1 = very dissatisfied; 5 = very satisfied) with free-text feedback collected.

**Results:**

During the evaluation period, no abnormal haematological results were recorded; all blood results remained within the green range. Two patients (6.25%) were withdrawn as aprecaution due to deterioration in physical health unrelated to blood monitoring frequency. There were no dropouts, no concerns regarding clinic attendance and all participants continued monthly physical health reviews. Mean patient satisfaction was 4.88/5, with feedback highlighting reduced anxiety, improved convenience and decreased distress related to venepuncture. Mean staff satisfaction was 4.96/5, with reported benefits including reduced patient distress, improved clinic efficiency and resource savings.

**Conclusion:**

In this pilot cohort, reducing long-term clozapine blood monitoring to three-monthly intervals was safe, well-tolerated and highly acceptable to both patients and staff when combined with ongoing monthly physical health reviews. This approach may reduce barriers to clozapine continuation, minimise invasive investigations and improve patient experience without compromising safety. Wider implementation and longer-term evaluation are warranted to support future service development and guideline review.

